# Macroscopic dynamics of the antiferroelectric smectic $Z_\textrm{A}$ phase and its magnetic analog $Z_\textrm{M}$

**DOI:** 10.1140/epje/s10189-025-00476-5

**Published:** 2025-02-27

**Authors:** Helmut R. Brand, Harald Pleiner

**Affiliations:** 1 Department of Physics, University of Bayreuth, 95440 Bayreuth, Germany; 2 Max Planck Institute for Polymer Research, 55021 Mainz, Germany

## Abstract

**Abstract:**

We analyze the macroscopic dynamics of antiferroelectric smectic $Z_\textrm{A}$ and antiferromagnetic smectic $Z_\textrm{M}$ liquid crystals. The smectic $Z_\textrm{A}$ phase is characterized by antiferroelectric order in one direction in the planes of the smectic layers giving rise to an orthogonal biaxial overall symmetry without polar direction. Thus in sufficiently thick (bulk) samples without externally applied electric fields, globally $D_{2h}$ symmetry results. Therefore, the macroscopic dynamics of the smectic $Z_\textrm{A}$ is isomorphic to that of the McMillan phase and one can take over the corresponding results in the field-free limit. This also applies to the defect structure in the sense that one can expect the appearance of half-integer defects as they have also been observed for the McMillan phase. Based on the fact that ferromagnetic nematic liquid crystals are known for about a decade, it seems natural to investigate the antiferromagnetic analog of the smectic $Z_\textrm{A}$ phase, which we denote as $Z_\textrm{M}$ in the present paper. In this phase, one also has an in-plane preferred direction, which is, however, not like a director in an ordinary nematic, but odd under time reversal. It can be characterized by a staggered magnetization, ${\varvec{N}}$, just as in a solid antiferromagnet like *MnO*. As additional macroscopic variables when compared to a usual non-polar smectic *A* phase, we have the in-plane staggered magnetization and the magnetization ${\varvec{M}}$. As a consequence, we find that spin waves (frequently called anti-magnons in solids) become possible. Therefore, we have for the antiferromagnetic smectic phase, $Z_\textrm{M}$, three pairs of propagating modes: first and ‘second’ sound as in usual smectic *A* phases and one pair of spin waves. The coupling between ‘second’ sound and spin waves is also analyzed leading to the possibility to excite spin waves by dynamic layer compressions and, vice versa, to generate ‘second’ sound by temporally varying magnetic fields. We note, however, that without additional mechanical or magnetic deformations, the coupling between spin waves on the one hand and first and second sound on the other is a higher order effect in the wave vector $\textbf{q}$. We also analyze the question of antiferroelectricity and antiferromagnetism for nematic liquid crystals.

**Graphical abstract:**

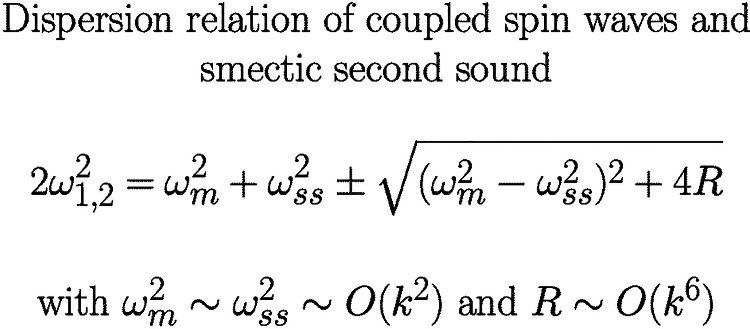

## Introduction

About two years ago, the experimental observation of polar smectic *A* phases has been announced in Refs. [1–3]. In these phases, which are orthogonal fluid smectic phases, the macroscopic polarization in the ground state is oriented parallel to the layer normal of the smectic planes and thus overall $C_{\infty v}$ symmetry prevails.

Another type of smectic phases with distinct electric properties is obtained, when the macroscopic polarization is in-plane, that is, perpendicular to the layer normal. Indeed, quite recently such a biaxial phase, the antiferroelectric smectic phase $Z_\textrm{A}$ has been experimentally found [4–8]. Here, the in-plane polarization flips the direction when going from one layer to the next, constituting an antiferroelectric phase.

The $Z_\textrm{A}$ phase is experimentally found between a usual (non-polar) nematic phase at higher temperatures and a ferroelectric (polar) nematic phase at lower temperatures in a number of compounds, [2–5, 9]. We mention that in Ref. [9] $Z_\textrm{A}$ was called $N_x$, since it was thought to be a nematic phase in this early paper, cf. also Sect. 4.

While ferroelectric nematic phases had been anticipated theoretically [10–12], this has apparently not been the case for ferroelectric smectic *A* phases. Over the last five years or so ferroelectric nematic phases have been investigated in detail, both experimentally [13–28] and theoretically [29–31].

On the magnetic side, ferromagnetic nematics are well established experimentally [32–38] and were also described theoretically [39–41]. However, ferromagnetic smectic phases are yet to be found. But stimulated by the experimental observation of ferroelectric smectic phases, it is tempting to investigate also ferromagnetic smectics: first, the orthogonal fluid smectic A phase, $A_M$, where the macroscopic magnetization in the ground state is oriented parallel to the layer normal (the magnetic analog to the ferroelectric smectic $A_F$ phase), and second, a possible antiferromagnetic smectic phase which we will call $Z_\textrm{M}$, where the macroscopic magnetic order is in-plane (perpendicular to the layer normal). Like the antiferroelectric analog, $Z_\textrm{A}$, the $Z_\textrm{M}$ phase can be expected to be antiferromagnetic rather than ferromagnetic.

It is the purpose of the present paper to characterize the antiferroelectric smectic *A* phase, $Z_\textrm{A}$, and the antiferromagnetic smectic *A* phase, $Z_\textrm{M}$, in the framework of macroscopic dynamics. These phases share the (orthogonal) layered structure of smectic *A*, but are different in the physical nature of the in-plane preferred direction that makes the systems biaxial: electric order in $Z_\textrm{A}$, and magnetic order in $Z_\textrm{M}$. In particular, we consider the ‘antiferro’ phases, where the directions of the in-plane preferred directions alternate from one layer to the next. As a result, we find that the two cases differ considerably with respect to the reversible, propagating modes. We also compare with the biaxial $C_M$ (‘McMillan’) phase [42–45], where the in-plane preferred direction is of nematic nature.

The $C_M$ phase shows a simple dielectric response to external electric fields [44]. This is qualitatively different from the response of the smectic $Z_\textrm{A}$ phase, because of the two sub-polarizations pointing into opposite directions. Various aspects of the field response to an external electric field have been studied in Refs. [9] and [3–5]. For example, hysteretic behavior as a function of an externally applied electric field is expected and has been observed. This has been elucidated in detail in Ref. [5] where it was demonstrated that $Z_\textrm{A}$ shows a double peak response, low permittivity and a polarization reversal current. We just mention that the dielectric response has been studied as a function of cell thickness and surface anchoring conditions [9].

To derive the macroscopic dynamic equations, we use the combination of irreversible thermodynamics and of symmetry arguments including the behavior of the macroscopic variables (and their associated currents and quasi-currents) under time reversal, parity (spatial inversion) as well as under rigid rotations and Galilei transformations [46–50]. This approach has been applied to many condensed matter systems including spin waves in magnets [50, 51], nematic liquid crystals [42, 46, 50, 52], ferroelectric smectic $A_F$ [53] and ferromagnetic $A_M$ [54], polymeric liquids [55–58] as well as superfluids including superfluid $^4$He [59, 60] and the superfluid phases of $^3$He [61–64].

The paper is organized as follows. In Sect. 2, we lay out the hydrodynamics of smectic $Z_\textrm{A}$ and $Z_\textrm{M}$. In Sect. 2.1, we discuss in detail our choice of the macroscopic variables. Due to the many structural similarities, we will present explicit formulas, for the free energy in Sect. 2.2, for the dynamic equations in Sect. 2.3, and for the dissipative currents in Sect. 2.4, only in terms of the $Z_\textrm{M}$ phase, but discuss the differences to $Z_\textrm{A}$ carefully. The physical nature of the in-plane directions, even under time reversal for $Z_\textrm{A}$ and odd under time reversal for $Z_\textrm{M}$, has a profound influence on the form of the reversible currents, which are therefore treated separately, Sects. 2.5 and 2.6 for $Z_\textrm{A}$ and $Z_\textrm{M}$, respectively. In Sect. 3, possible static as well as dynamic experiments for the antiferromagnetic phase as well as for the antiferroelectric phase are outlined to test our predictions. In Sect. 4, the question of the possibility to have antiferromagnetic or antiferroelectric nematic phases is examined. In Sect. 5, we analyze the possible defects in the antiferroelectric $Z_\textrm{A}$ phase also including an analysis of recent experimental reports on the defects observed in the $Z_\textrm{A}$ phase. A brief summary and a perspective are given in Sect. 6.

## Hydrodynamics of the $Z_\textrm{A}$ and the $Z_\textrm{M}$ phase

### Symmetry properties and macroscopic variables ($Z_\textrm{A}$ and $Z_\textrm{M}$)

Making use of the properties of the ground state, we can deduce the macroscopic variables for the description of the smectic $Z_\textrm{M}$ and $Z_A$ phase. As in simple liquids, there are the conserved quantities, mass density, $\rho$, energy density, $\varepsilon$, density of linear momentum, $g_i$, and the case of mixtures, the concentration, $\phi$, as macroscopic variables.

The smectic *A* layer ordering allows a translation $u_i$ along the layer normal $k_i$ as Goldstone mode, leading to $u = k_i u_i$ as hydrodynamic variable. It is a scalar variable, but subject to the $k_i \rightarrow -k_i$ invariance ($I_k$) that is similar to the familiar $n_i \rightarrow -n_i$ invariance for nematic directors. Deformations and rotations of the smectic structure are described by gradients of *u*.

The in-plane preferred direction in the $Z_\textrm{M}$ phase is described by a staggered magnetization

1 $$\begin{aligned} N_i = \sum _{n=1}^{n_{\textrm{max}}} \varDelta _n M_i^n \end{aligned}$$

with $\varDelta _n =1$ for odd-numbered layers and $\varDelta _n =-1$ for even ones. $M_i^n$ is the magnetization in the n-th layer. Of course, the choice what is called an ‘odd’ or ‘even’ layer is arbitrary, and the opposite choice for $\varDelta$ leads to $-N_i$ as staggered magnetization, at least for $n_{\textrm{max}} \gg 1$ (so-called thick sample). The definition of the staggered magnetization in Eq. (1) parallels closely that used by refs. [50, 51] in the context of antiferromagnetism. It is the staggered analog of the magnetization. Thus, $<N_i> = N_0 {\hat{z}}$ is the analog of the spontaneous magnetization in a ferromagnet: $<M_i> = M_0 {\hat{z}}$ if we take the $z-$ direction as the arbitrary preferred direction. In the case considered here for antiferromagnetic smectic *A*, this preferred direction is an arbitrary direction in the layer planes, which we take to be the $x-$ direction and which is perpendicular to the layer normal. The normalized preferred direction is denoted by ${\hat{N}}_i$. And the broken symmetry is then associated with rotations of ${\hat{N}}_i$ in the layer planes.

Therefore, all equations have to obey a $N_i \rightarrow -N_i$ invariance ($I_N$). As a result, the in-plane preferred direction $N_i$ (and its normalized version ${\hat{N}}_i$) is odd under $I_N$, even under spatial inversion $\epsilon _S$ (an axial vector), and odd under time inversion, $\epsilon _T$. The last two properties are the same as for a magnetization. Biaxiality is manifested by the three mutually orthogonal unit vectors ${\hat{k}}_i$, ${\hat{N}}_i$, and

2 $$\begin{aligned} {\hat{l}}_i \equiv \epsilon _{ijk} {\hat{k}}_j {\hat{N}}_k. \end{aligned}$$

Rotations of $N_i$ within the smectic layers are the Goldstone mode giving rise to the hydrodynamic variable

3 $$\begin{aligned} \delta N = {\hat{l}}_i \delta N_i \end{aligned}$$

which is a scalar variable subject to $I_k$.

Although there is no net magnetization (no ferromagnetism) $M_i =0$ in the equilibrium structure due to the staggered magnetization structure, we keep $M_i$ as a variable. It is not related to a broken symmetry. It is an axial vector and odd under $\epsilon _T$.

**Fig. 1:**
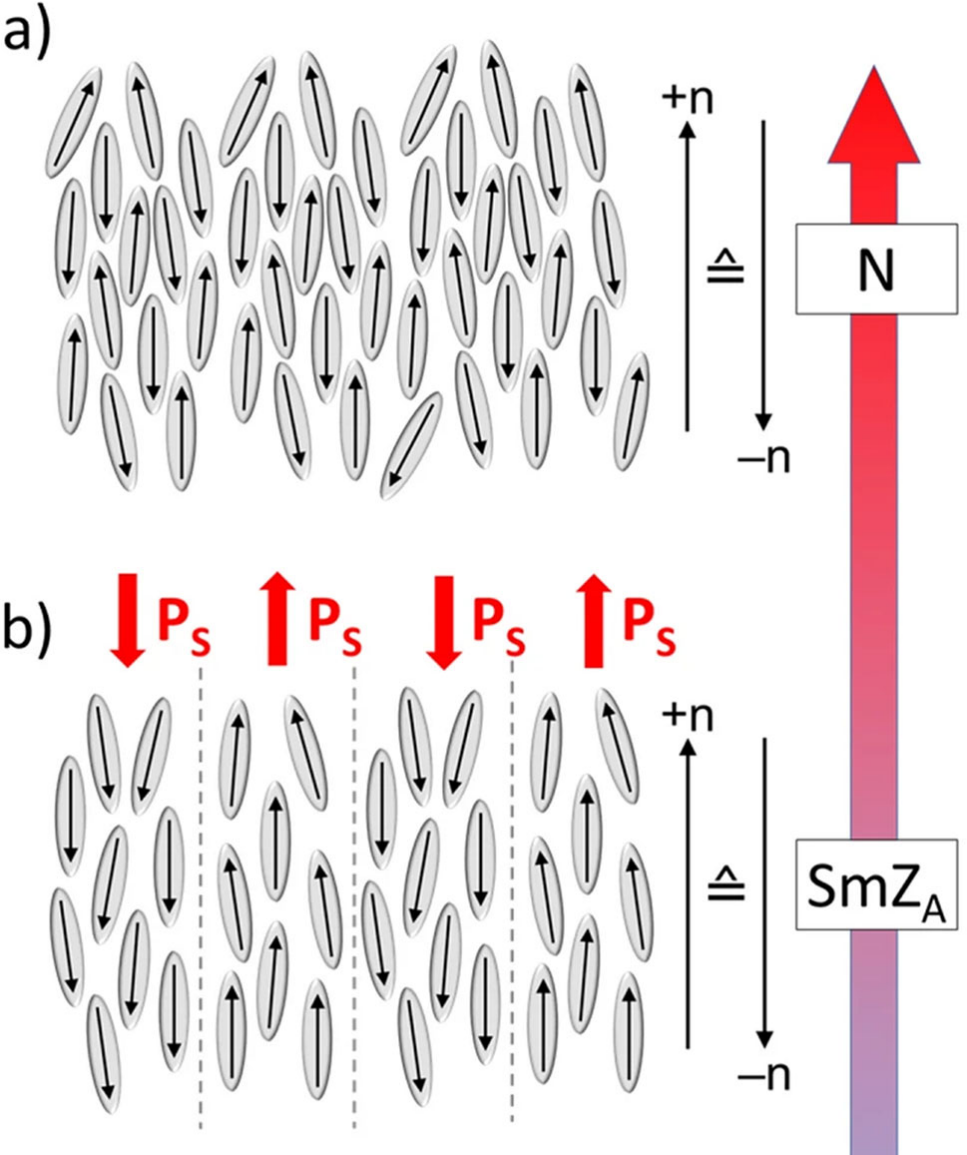
**a** A typical nematic phase is shown, which is disordered positionally in three dimensions. There is no net electric polarization, and one has $n_i \rightarrow - n_i$ symmetry. **b** In the smectic $Z_\textrm{A}$ phase, a layered structure arises. The director field is oriented parallel to the layering in contrast to a usual smectic *A* phase. The electric polarization changes sign from one layer to the next, and the layer normal ${\hat{k}}_i$ is oriented perpendicular to the director field as well as to the electric polarization. Therefore, a staggered electric polarization $Q_i$ results. (Adapted from Ref. [5].)

For the $Z_\textrm{A}$ phase plotted in Fig. 1, one gets a similar set of variables as discussed above, when one replaces the staggered magnetization by the staggered polarization

4 $$\begin{aligned} Q_i = \sum _{n=1}^{n_{\textrm{max}}} \varDelta _n P_i^n \end{aligned}$$

which is subject to the $I_Q$ invariance, $Q_i \rightarrow -Q_i$, and is odd under spatial inversion $\epsilon _S$ (polar vector), and even under time inversion, $\epsilon _T$, just like $P_i$. It gives rise to the Goldstone variable

5 $$\begin{aligned} \delta Q = \epsilon _{ijk} {\hat{k}}_j {\hat{Q}}_k \delta Q_i \end{aligned}$$

which, like $\delta N$, is subject to $I_k$. The orthogonal triad is ${\hat{k}}_i$, ${\hat{Q}}_i$, and $\epsilon _{ijk} {\hat{k}}_j {\hat{Q}}_k$. Compared to $Z_\textrm{M}$, one has to replace $M_i$ by $P_i$ as variable in $Z_\textrm{A}$.

Here, it is worthwhile to compare with the McMillan phase $C_M$, which is a biaxial smectic A phase [44]. The in-plane preferred direction ${\hat{m}}_i$, (not to be mixed up with the magnetization $M_i$), is of nematic nature with a $m_i \rightarrow -m_i$ invariance ($I_m$). In the $C_M$ phase, $I_k$ and $I_m$ are independent invariances. The hydrodynamic variable is $\delta m = \epsilon _{ijk} {\hat{k}}_j {\hat{m}}_k \delta m_i$.

The symmetry properties of the three Goldstone variables $\delta m$, $\delta N$ and $\delta Q$, are identical, but the time reversal behavior of the in-plane preferred directions is different, odd for ${\hat{N}}_i$ and even for ${\hat{Q}}_i$ and ${\hat{m}}_i$. As a result, the $Z_\textrm{A}$ and the $C_M$ phase can be expected to be rather similar (without external fields), while the $Z_\textrm{M}$ phase will differ from them, in particular for the reversible currents.

Assuming local thermodynamic equilibrium, i.e., all fast relaxing quantities being already in equilibrium, the local formulation of the first law of thermodynamics, including the magnetic induction ${{\varvec{B}}}$, reads [46–48]

6 $$\begin{aligned} d\varepsilon= &   T d \sigma +\mu d \rho +{ \varPi } d \phi + v_i d g_i + H_i d B_i \nonumber \\  &   \quad +h_i^{M} d M_i + h_i^N d \nabla _i N + h^u_i d \nabla _i u \end{aligned}$$

connecting the macroscopic variables to the entropy density $$\sigma $$. In Eq. (6), the thermodynamic quantities: chemical potential ($$\mu $$), temperature (*T*), relative chemical potential ($${ \varPi }$$), the molecular field $$ h^u_i$$ associated with the layer displacement *u*, the magnetic Maxwell field ($$H_i$$), the magnetic molecular field $$h_i^M$$ and the molecular field $$h_i^N$$, associated with the staggered magnetization in the layer planes, are defined as partial derivatives of the energy density with respect to the appropriate variables [47]. Since the free energy has to be a scalar, it is invariant under spatial inversions, time inversion, $$I_k$$ and $$I_N$$ invariance. The conjugate quantities inherit the symmetry properties of the variables.

For the $Z_\textrm{A}$ phase, Eq. (6) is replaced by

7 $$\begin{aligned} \textrm{d}\varepsilon= &   T \textrm{d} \sigma +\mu \textrm{d} \rho +{ \varPi } \textrm{d} \phi + v_i \textrm{d} g_i + E_i \textrm{d} D_i \nonumber \\  &   \quad +h_i^{P} \textrm{d} P_i + h_i^Q \textrm{d} \nabla _i Q + h^u_i \textrm{d} \nabla _i u \end{aligned}$$

with the molecular fields $$h_i^Q$$ and $$h_i^P$$ (instead of $$h_i^N$$ and $$h_i^M)$$ and $$\varepsilon $$ has to be invariant under $$I_Q$$ invariance (instead of $$I_N$$).

### Static considerations ($Z_\textrm{A}$ and $Z_\textrm{M}$)

First we analyze the static properties of smectic $Z_\textrm{M}$ without taking into account the static Maxwell equations in the magnetic domain. Field effects will be discussed later in this section.

The static behavior of the macroscopic system studied here is conveniently described by the energy functional in harmonic approximation, Ref. [11, 46, 47, 65]. We obtain, including the kinetic energy density

8 $$\begin{aligned} \varepsilon= &   \frac{1}{2} \chi _{ij}^M M_i M_j + \frac{1}{2}L_{ij}^N(\nabla _i N)(\nabla _j N) \nonumber \\  &   \quad + \frac{1}{2} B (\nabla _z u)^2 + \frac{1}{2} A_{ijkm} (\nabla _i \nabla _j u) (\nabla _k \nabla _m u) \nonumber \\  &   \quad + D_{ijk} (\nabla _i \nabla _j u) (\nabla _k N) {+ \frac{1}{2} {\tilde{A}}_{ijkm}(\nabla _i \nabla _j u) (\nabla _k \nabla _m u)} \nonumber \\  &   \quad + \frac{1}{2}c_{\rho \rho } (\delta \rho )^2 + \frac{1}{2}c_{\sigma \sigma }(\delta \sigma )^2 + \frac{1}{2}c_{\phi \phi }(\delta \phi )^2 \nonumber \\  &   \quad + c_{\rho \phi }(\delta \rho )(\delta \phi ) + c_{\rho \sigma }(\delta \rho )(\delta \sigma ) +c_{\sigma \phi }(\delta \sigma )(\delta \phi ) \nonumber \\  &   \quad + (\theta _1 \delta \rho + \theta _2 \delta \sigma + \theta _3 \delta \phi ) \, \nabla _z u \nonumber \\  &   \quad + \frac{1}{2} K_{ijkl}^{M} (\nabla _i M_j) (\nabla _k M_l) + \frac{1}{2\rho } {\varvec{g}}^2 \end{aligned}$$

Since *u* and $\delta N$ are Goldstone modes, only gradients of these quantities can enter the free energy functional. The gradient $\nabla _z$ is a short hand notation for ${\hat{k}}_i \nabla _i$. A ‘$\delta$’ denotes deviations from the equilibrium value, in particular $\delta \phi = \phi - \phi _0$, $\delta \rho = \rho - \rho _0$, and $\delta \sigma = \sigma - \sigma _0$.

The second, third and fourth rank properties tensors listed in Eq. (8) reflect the biaxial orthorhombic symmetry of the antiferromagnetic smectic $Z_\textrm{M}$ phase. The second rank property tensors $\chi _{ij}^M$ and $L_{ij}^N$ take the form

9 $$\begin{aligned} T_{ij} = T_1 {\hat{k}}_i {\hat{k}}_j + T_2 {\hat{N}}_i {\hat{N}}_j + T_3 {\hat{l}}_i {\hat{l}}_j \end{aligned}$$

The third rank $D_{ijk}$ coupling second gradients of the layer displacement *u* to gradients of $\delta N$ is of the form

10 $$\begin{aligned} D_{ijk}= &   \frac{1}{6} D( {\hat{l}}_i {\hat{N}}_j {\hat{k}}_k + {\hat{l}}_i {\hat{N}}_k {\hat{k}}_j + {\hat{l}}_j {\hat{N}}_i {\hat{k}}_k \nonumber \\  &   \quad + {\hat{l}}_j {\hat{N}}_k {\hat{k}}_i + {\hat{l}}_k {\hat{N}}_i {\hat{k}}_j + {\hat{l}}_k {\hat{N}}_j {\hat{k}}_i ) \end{aligned}$$

This third rank tensor thus has only one independent coefficient bringing into play gradients in all three different orthogonal directions of our biaxial system. It couples, for example, layer compressions to in-plane distortions of the preferred direction in the layer planes. It assumes the same structure as the analogous term in the hydrodynamics of smectic $C_M$ [44].

The fourth rank tensor $K_{ijkl}^M$ has the same form as the Frank elastic tensor of orthorhombic biaxial nematics [66, 67] and has therefore twelve bulk elastic coefficients [68] and three surface ones [69].

Finally, the fourth rank tensor describing in-plane deformations of the displacement *u* takes in our biaxial system the form

11 $$\begin{aligned} A_{ijkm}= &   A_1 {\hat{l}}_i {\hat{l}}_j {\hat{l}}_k {\hat{l}}_m + A_2 {\hat{N}}_i {\hat{N}}_j {\hat{N}}_k {\hat{N}}_m + A_3 ( {\hat{l}}_i {\hat{l}}_j {\hat{N}}_k {\hat{N}}_m \nonumber \\  &   \quad + {\hat{l}}_i {\hat{l}}_k {\hat{N}}_j {\hat{N}}_m +{\hat{l}}_i {\hat{l}}_m {\hat{N}}_k {\hat{N}}_j +{\hat{l}}_k {\hat{l}}_j {\hat{N}}_i {\hat{N}}_m \nonumber \\  &   \quad + {\hat{l}}_m {\hat{l}}_j {\hat{N}}_i {\hat{N}}_k +{\hat{l}}_k {\hat{l}}_m {\hat{N}}_i {\hat{N}}_j ) \end{aligned}$$

obtaining three independent coefficients in the present biaxial system, while in the uniaxial smectic *A* there is one (usually called *K*).

Terms of the structure $C_{ijkl} (\nabla _i \nabla _j u) (\nabla _l M_k)$, $C_{ijk} (\nabla _i N) (\nabla _j M_k)$ and $C_{ij} M_i (\nabla _j N)$ do not arise in the energy density expansion, since they are not compatible with the requirement that the energy density is a good scalar under all symmetry operations of the system including parity, time reversal, $k_i \rightarrow - k_i$ invariance and $N_i \rightarrow - N_i$ invariance.

To guarantee stability of the energy density for the contribution $\sim D_{ijk}$, we have added in Eq. (8) in the third line the contribution $\frac{1}{2} {\tilde{A}}_{ijkm}(\nabla _i \nabla _j u) (\nabla _k \nabla _m u)$ to include out-of-plane gradients, where ${\tilde{A}}_{ijkm}$ takes the form

12 $$\begin{aligned} {\tilde{A}}_{ijkm}  &   = {\tilde{A}}_1 ( {\hat{k}}_i {\hat{k}}_j {\hat{N}}_k {\hat{N}}_m + {\hat{k}}_i {\hat{k}}_k {\hat{N}}_j {\hat{N}}_m +{\hat{k}}_i {\hat{k}}_m {\hat{N}}_k {\hat{N}}_j \nonumber \\  &   \quad +{\hat{k}}_k {\hat{k}}_j {\hat{N}}_i {\hat{N}}_m + {\hat{k}}_m {\hat{k}}_j {\hat{N}}_i {\hat{N}}_k +{\hat{k}}_k {\hat{k}}_m {\hat{N}}_i {\hat{N}}_j ) \nonumber \\  &   \quad + {\tilde{A}}_2 ( {\hat{k}}_i {\hat{k}}_j {\hat{l}}_k {\hat{l}}_m + {\hat{k}}_i {\hat{k}}_k {\hat{l}}_j {\hat{l}}_m +{\hat{k}}_i {\hat{k}}_m {\hat{l}}_k {\hat{l}}_j +{\hat{k}}_k {\hat{k}}_j {\hat{l}}_i {\hat{l}}_m \nonumber \\  &   \quad + {\hat{k}}_m {\hat{k}}_j {\hat{l}}_i {\hat{l}}_k +{\hat{k}}_k {\hat{k}}_m {\hat{l}}_i {\hat{l}}_j ) + {\tilde{A}}_3 ( {\hat{k}}_i {\hat{k}}_j {\hat{k}}_k {\hat{k}}_m) \end{aligned}$$

Gradients of *u* describing compression/dilatation ($\sim B$) are unchanged when compared to uniaxial smectic *A*.

In addition, the energy density of a fluid binary mixture, involving $\delta \sigma$, $\delta \rho$ and $\delta \phi$, is as in the non-magnetic case.

A positive-definite free energy is necessary to guarantee static stability of system. This is obtained by the conditions $$ B > 0 $$, $$L_1^N > 0$$, $$L_2^N > 0$$, $$L_3^N > 0$$, $$ 36 L_1^N A_3 > D^2$$, $$36 L_2^N {\tilde{A}}_1 > D^2$$, and $$ 36 L_1^N {\tilde{A}}_2 > D^2$$, as well as $$\chi _1^M > 0$$, $$\chi _2^M > 0$$ and $$\chi _3^M > 0$$. Although the energy density Eq. (8) is bilinear in deviations from equilibrium, there are intrinsic nonlinear effects, since the material parameters can be functions of the (scalar) state variables, like temperature and density.

The harmonic approximation is a restriction to sufficiently small deviations from the spatially homogeneous ground state. The nonlinear correction, e.g., due to the replacement $\nabla _z u \rightarrow \nabla _z u - \frac{1}{2} ({\varvec{\nabla }}_{\perp } u )^2$ in Eq. (8), takes care of effective layer compressions due to rotations as is explained in Fig. 3b of Ref. [70].

In contrast to a ferromagnetic direction, the staggered magnetization $N_i$ is not oriented linearly by an external field, but only by the diamagnetic anisotropy effect, which is quadratic in the field strength. Adding to Eq. (8), the magnetic energy $\varepsilon ^H = - (1/2) (\chi ^H_{ij})^{-1} B_i B_j = - (1/2) \chi ^H_{ij} H_i H_j$ with the material tensors of the form Eq. (9); the anisotropic parts

13 $$\begin{aligned} 2 \varepsilon ^H_a= &   - (\chi ^H_1 - \chi ^H_3) (H_i {\hat{k}}_i)^2 - (\chi ^H_2 - \chi ^H_3) (H_i {\hat{N}}_i)^2\nonumber \\ \end{aligned}$$

govern the orientation of the field relative to the smectic structure.

Stable cases are, e.g., $H_i \parallel {\hat{k}}_i$ and $H_i \perp {\hat{N}}_i$ for $\chi ^H_1 > \chi ^H_3$ and $\chi ^H_2 < \chi ^H_3$, while for the opposite case $\chi ^H_1 < \chi ^H_3$ and $\chi ^H_2 > \chi ^H_3$ the field is parallel to the in-plane direction and perpendicular to the layer normal.

Similar considerations apply for external electric fields, The simple relation for the dielectric energy $\varepsilon ^E = - (1/2) (\chi ^E_{ij})^{-1} D_i D_j = - (1/2) \chi ^E_{ij} E_i E_j$, however, can only be used, if the flexoelectric energy $\varepsilon ^{fl} = \xi _{ij}^{fl} D_i \nabla _j N$ is neglected [47].

According to the Gibbs relation, Eq. (6), the conjugate quantities of the $Z_\textrm{M}$ phase follow from the free energy as variational or partial derivatives with respect to the appropriate variable, while all the other variables are kept constant.

14 $$\begin{aligned} h_i^{\prime u}= &   \frac{\partial \varepsilon }{\partial \nabla _i u} \big \arrowvert _{\dots } = {\hat{k}}_i (B \nabla _z u + \theta _1 \delta \rho + \theta _2 \delta \sigma + \theta _3 \delta \phi )\nonumber \\ \end{aligned}$$

15 $$\begin{aligned} \varPhi _{ij}^u= &   \frac{\partial \varepsilon }{\partial \nabla _i \nabla _j u} \big \arrowvert _{\dots } = A_{ijkm} (\nabla _k \nabla _m u) + D_{ijk} (\nabla _k N) \nonumber \\  &   \hspace{2cm} {+ {\tilde{A}}_{ijkm} (\nabla _k \nabla _m u)} \end{aligned}$$

16 $$\begin{aligned} h_i^{\prime M} \!= &   \frac{\partial \varepsilon }{\partial M_i} \big \arrowvert _{\dots } = \chi _{ij}^M M_j \end{aligned}$$

17 $$\begin{aligned} \varPhi ^{M}_{ij}= &   \frac{\partial \varepsilon }{\partial (\nabla _j M_i)}\big \arrowvert _{\dots } = K_{ijkl}^{M} (\nabla _l M_k) \end{aligned}$$

18 $$\begin{aligned} h_i^N= &   \frac{\partial \varepsilon }{\partial (\nabla _i N)}\big \arrowvert _{\dots } = L_{ij}^N (\nabla _j N) + D_{jik} (\nabla _k \nabla _j u) \end{aligned}$$

19 $$\begin{aligned} \delta \mu= &   \frac{\partial \varepsilon }{\partial \delta \rho }\big \arrowvert _{\dots } = \theta _1 \nabla _z u + c_{\rho \rho }\delta \rho + c_{\rho \phi }\delta \phi + c_{\rho \sigma }\delta \sigma \end{aligned}$$

20 $$\begin{aligned} \delta T= &   \frac{\partial \varepsilon }{\partial \delta \sigma }\big \arrowvert _{\dots } = \theta _2 \nabla _z u + c_{\sigma \sigma }\delta \sigma + c_{\rho \sigma }\delta \rho + c_{\sigma \phi }\delta \phi \qquad \end{aligned}$$

21 $$\begin{aligned} \delta { \varPi }= &   \frac{\partial \varepsilon }{\partial \delta \phi }\big \arrowvert _{\dots } = \theta _3 \nabla _z u + c_{\phi \phi }\delta \phi + c_{\rho \phi }\delta \rho + c_{\sigma \phi }\delta \sigma \end{aligned}$$

22 $$\begin{aligned} v_i= &   \frac{\partial \varepsilon }{\partial g_i} \big \arrowvert _{\dots } = g_i/\rho \end{aligned}$$

where the ellipses $${\dots }$$ indicate that all other quantities—except for the one associated with the derivative taken—are fixed. For the variables $$ M_i$$ and $$\nabla _i u$$, which enter the energy at different gradient levels, we have split the conjugates $$h_i^M = h_i^{'M} - \nabla _j\varPhi ^M_{ij} $$ and $$h_i^u = h_i^{'u} - \nabla _j\varPhi ^u_{ij} $$.

This section can be taken over for $Z_\textrm{A}$, if $M_i$ is replaced by $P_i$, ${\hat{N}}_i$ by ${\hat{Q}}_i$ and ${\hat{l}}_i$ by ${\hat{l}}_i^Q = \epsilon _{ijk} {\hat{k}}_j {\hat{Q}}_k$.

### Dynamics ($Z_\textrm{A}$ and $Z_\textrm{M}$)

In the $Z_\textrm{M}$ phase, the hydrodynamic equations for conserved, broken symmetry and slowly relaxing variables are

23 $$\begin{aligned}  &   {\dot{\rho }} + \nabla _i (\rho v_i) = 0, \end{aligned}$$

24 $$\begin{aligned}  &   {\dot{\sigma }} + \nabla _i (\sigma v_{i} + j_i^{\,\sigma {\mathrm R}} + j_i^{\,\sigma {\mathrm D}}) = \frac{2R}{T}, \end{aligned}$$

25 $$\begin{aligned}  &   \dot{g}_i + \nabla _j (g_i v_j + p \, \delta _{ij} - {\hat{k}}_i h_j^u + \sigma _{ij}^{\textrm{th}} + \sigma _{ij}^{\, \textrm{R}} + \sigma _{ij}^{\,\textrm{D}} ) = 0,\nonumber \\ \end{aligned}$$

26 $$\begin{aligned}  &   {\dot{\phi }} + v_j \nabla _j \phi + X^{\phi \textrm{R}} + X^{\phi \textrm{D}} + \nabla _i ( j_i^{\phi \textrm{R}} + j_i^{\phi \textrm{D}}) = 0, \end{aligned}$$

27 $$\begin{aligned}  &   \dot{u} + v_j \nabla _j u -v_z+ X^{u \textrm{R}} + X^{u \textrm{D}} = 0, \end{aligned}$$

28 $$\begin{aligned}  &   \dot{N} + v_i \nabla _i N + X^{N \textrm{R}} + X^{N \textrm{D}} = 0, \end{aligned}$$

29 $$\begin{aligned}  &   {\dot{M}}_i +v_{j}\nabla _{j} M_{i}+\left( {{\varvec{M}}} \times {\varvec{\omega }}\right) _i \nonumber \\  &   \quad + \nabla _j (j_{ij}^{M \textrm{R}} + j_{ij}^{M \textrm{D}} )=0, \end{aligned}$$

where a dot on top of a variable means a partial derivative $\partial / \partial t$ with respect to time and where $\omega _i=\frac{1}{2}\epsilon _{i j k}\nabla _j v_k$ is the vorticity. The conserved quantities and the entropy density contain the divergence of phenomenological currents, while quasi-currents (without a divergence) are associated either with spontaneously broken continuous symmetry variables or with other macroscopic variables. In particular, Eq. (28) describes the dynamics of the spontaneously broken rotational symmetry in the layer planes associated with the staggered magnetization. For the concentration variable, we have allowed for both possibilities, conserved or non-conserved. Only for $X^{\phi \textrm{R}} + X^{\phi \textrm{D}} = 0$ is $\delta \phi$ conserved.

The superscripts *D* and *R* on the currents denote, respectively, the dissipative and reversible parts.

We use the pressure *p* including the isotropic part of the Maxwell stress

30 $$\begin{aligned} p = - \frac{\partial \,( \int \!\varepsilon \textrm{d}V )}{ \partial V}= -\varepsilon + \mu \rho + T\sigma + {\varvec{ v\cdot g}} + B_i H_i \end{aligned}$$

and the off-diagonal terms of the Maxwell- and the Ericksen-type stresses [71]

31 $$\begin{aligned} 2 \sigma ^{\textrm{th}}_{ij}= &   - \left( H_iB_j + B_iH_j\right) + \varPhi _{li}^M \nabla _j M_l + \varPhi _{lj}^M \nabla _i M_l \nonumber \\    &   + \varPhi _{ki}^u \nabla _k \nabla _{j} u + \varPhi _{kj}^u \nabla _k \nabla _i u \end{aligned}$$

The Maxwell stress is of the standard form [49, 72] with $B_i = \mu _0 (H_i + M_i)$ and has been symmetrized with the help of the requirement that the energy density should be invariant under rigid rotations [46, 47].

The entropy production 2*R*/*T* in Eq. (24) is a measure for the energy dissipation of the system. Due to the second law of thermodynamics, the dissipation *R* must satisfy $\int R \, dV \ge 0$: For irreversible processes *R* is positive, and for reversible ones it is equal to zero (or a total divergence)

32 $$\begin{aligned} 2 R= &   -j_i^{\sigma *} \nabla _i T + X^{\phi *} { \varPi } - j_{i}^{\phi *} \nabla _{i} { \varPi } - \sigma _{ij}^{*} \nabla _j v_i \nonumber \\  &   {-} j_{ij} ^{M*} \nabla _j h_i^M - X^{u *} \nabla _i h_i^u - X^{N*} \nabla _i h^N_i \ge 0\qquad \end{aligned}$$

where the upper sign applies to $* = \textrm{D}$ and the lower one to $*=\textrm{R}$.

The energy conservation law

33 $$\begin{aligned} {\dot{\varepsilon }} + \nabla _i (\varepsilon + p) \,v_i + \nabla _i \bigl ( j_i^{\,\varepsilon {\textrm{R}}} + j_i^{\,\varepsilon {\textrm{D}}} \bigr ) = 0 , \end{aligned}$$

is redundant due to the Gibbs relation, Eq. (7), and follows from Eqs. (23)-(32). In particular, $j_i^{\,\varepsilon {\textrm{D}} }= T j_i^{\,\sigma {\mathrm D}} +{ \varPi } j_i^{\phi \textrm{D}} +v_j \sigma _{ij}^{\,\textrm{D}} +h_j^M j_{ij}^{M \textrm{D}} + h_i^u X^{u \textrm{D}}$.

The phenomenological currents and quasi-currents are the sum of the reversible and the dissipative part, as can be seen in Eqs. (24)–(29). The various transport contributions in Eqs. (23)–(29) (as well as *p* and $\sigma ^{\textrm{th}}_{ij}$) are reversible and add up to zero in the entropy production. They are not material dependent, but are given by general symmetry and thermodynamic principles [47], like transformation behavior under translations (convective terms) or rotations (e.g., $\ldots \times {\varvec{\omega }}$).

These phenomenological currents and quasi-currents are treated in the following sections within ‘linear irreversible thermodynamics’ (guaranteeing general Onsager relations), i.e., as linear relations between currents and thermodynamic forces. The resulting expressions are nevertheless nonlinear, since all material parameters can be functions of the scalar state variables (e.g., $\sigma$, $\rho$, $\phi$).

This section can be taken over for $Z_\textrm{A}$, if $M_i$ is replaced by $P_i$ and $j_{ij}^{M {\textrm{R}}}$, $j_{ij}^{M {\textrm{D}}}$ by $j_{ij}^{P {\textrm{R}}}$, $j_{ij}^{P {\textrm{D}}}$.

### Dissipative currents ($Z_\textrm{A}$ and $Z_\textrm{M}$)

For the derivation of the dissipative parts of the phenomenological currents, one usually expands the dissipation function *R* to second order in the thermodynamic forces and then obtains the dissipative currents by taking the variational derivatives with respect to the forces. We find for the dissipation function of the $Z_\textrm{M}$ phase

34 $$\begin{aligned} 2 R= &   \kappa _{i j}\left( \nabla _{i} T\right) \left( \nabla _{j} T\right) + D_{ij} (\nabla _i { \varPi })(\nabla _j { \varPi }) \nonumber \\  &   \quad + 2D^{T { \varPi }}_{ij} (\nabla _i T)(\nabla _j { \varPi }) + {\tilde{\mu }} (\delta { \varPi })^2 \quad \nonumber \\  &   \quad + b_{ijkl} (\nabla _j h_i^M)(\nabla _l h_k^M )+ \nu _{ijkl} A_{ij}A_{kl} \nonumber \\    &   \quad + 2 D^{u{ \varPi }} (\nabla _z { \varPi }) (\nabla _k h_k^u) + 2 D^{uT} (\nabla _z T) (\nabla _i h_i^u) \nonumber \\    &   \quad + \frac{1}{\gamma ^N} (\nabla _i h^N_i)^2 + \frac{1}{\gamma _u} (\nabla _i h_i^u)^2 \end{aligned}$$

Here, $\nu _{ijkl}$ is the biaxial viscosity tensor for orthorhombic systems [66, 68] with nine independent coefficients, while the symmetric 2nd rank tensors $\kappa _{ij}$, $D_{ij}$ and $D^T_{ij}$ heat conduction, diffusion and thermodiffusion, respectively, have three independent coefficients each and have the form given in Eq. (9). Smectic permeation is given by one coefficient $1/\gamma _{u}$. The same applies to the diffusive processes as associated with the variable $\delta N$ characterizing the spontaneously broken rotational symmetry in the smectic planes. Diffusive processes associated with the magnetization are contained in the contribution with the fourth rank tensor $b_{ijkl}$. Due to its orthorhombic symmetry, it has 12 independent coefficients [68]. Concentration relaxation is governed by ${\tilde{\mu }}$; for a conserved concentration variable, ${\tilde{\mu }} = 0$.

The range of possible values of the coefficients in Eq. (34) is restricted by the positivity of the entropy production that requires, e.g., $D_{1} / \gamma _u > (D^{u { \varPi }})^2$, $\kappa _{1} /\gamma _u > (D^{u T})^2$, $\gamma ^N$ and $\gamma _u$ all positive.

To obtain the dissipative parts of the currents and quasi-currents, we take the partial derivatives of *R* with respect to the appropriate thermodynamic force

35 $$\begin{aligned} j^{\sigma \textrm{D}}_i = - \frac{\partial R}{\partial (\nabla _i T)}\big \arrowvert _{\dots }= &   - \kappa _{ij} \nabla _j T - D_{ij}^{T { \varPi }} \nabla _j { \varPi } \nonumber \\    &   - D^{uT} {\hat{k}}_i \nabla _j h_j^u \end{aligned}$$

36 $$\begin{aligned} j^{\phi \textrm{D}}_i = - \frac{\partial R}{\partial (\nabla _j{ \varPi })}\big \arrowvert _{\dots }= &   - D_{ij} \nabla _j{ \varPi } - D^{T{ \varPi }}_{ij} \nabla _j T \nonumber \\  &   - D^{u{ \varPi }} {\hat{k}}_i \nabla _j h_j^u \end{aligned}$$

37 $$\begin{aligned} X^{\phi \textrm{D}} = \frac{\partial R}{\partial { \varPi }}\big \arrowvert _{\dots }= &   {\tilde{\mu }} \,\delta { \varPi } \end{aligned}$$

38 $$\begin{aligned} \sigma ^{\textrm{D}}_{ij} = - \frac{\partial R}{\partial A_{ij} }\big \arrowvert _{\dots }= &   - \nu _{ijkl}A_{kl} \end{aligned}$$

39 $$\begin{aligned} X^{N \textrm{D}} = - \frac{\partial R}{\partial \nabla _i h^N_i}\big \arrowvert _{\dots }= &   - \frac{1}{\gamma ^N} \nabla _i h^N_i \end{aligned}$$

40 $$\begin{aligned} j^{M \textrm{D}}_{ij} = - \frac{\partial R}{\partial (\nabla _j h^M_i) }\big \arrowvert _{\dots }= &   - b_{ijkl} \nabla _k h_l^M \end{aligned}$$

41 $$\begin{aligned} X^{u \textrm{D}} = - \frac{\partial R}{\partial (\nabla _i h_i^u) }\big \arrowvert _{\dots }= &   - \frac{1}{\gamma _u} \nabla _i h_i^u - D^{u{ \varPi }} \nabla _z { \varPi } \nonumber \\  &   - D^{uT} \nabla _z T \end{aligned}$$

### Reversible currents for $Z_\textrm{M}$

The material tensors with a superscript *R* have to be odd under time reversal and therefore must contain an odd number of ${\hat{N}}_i$ factors. In addition, they are even in ${\hat{k}}_i$. As a result, they are subject to $I_N$ invariance (${\hat{N}}_i \rightarrow -{\hat{N}}_i$) and cannot connect simple variables, like $T,\varPi ,\phi ,\sigma$ or $v_i, g_i$. Only cross-couplings involving $\delta N$ or its conjugate or its quasi-current are possible.

The reversible phenomenological currents, with superscript R in Eqs. (23)–(29), give rise to zero entropy production, Eq. (32). Taking into account the symmetry behavior regarding space inversion and time reversal as well as under ${\hat{k}}_i \rightarrow -{\hat{k}}_i$ invariance, we obtain

42 $$\begin{aligned} j_{i}^{\sigma \textrm{R}}= &   0 \end{aligned}$$

43 $$\begin{aligned} j_{i}^{\phi \textrm{R}}= &   0 \end{aligned}$$

44 $$\begin{aligned} X^{\phi \textrm{R}}= &   0 \end{aligned}$$

45 $$\begin{aligned} \sigma _{ij}^{\textrm{R}}= &   - \lambda _{ji}^N \nabla _k h_k^N \end{aligned}$$

46 $$\begin{aligned} X^{u \textrm{R}}= &   0 \end{aligned}$$

47 $$\begin{aligned} j_{ij}^{M \textrm{R}}= &   \gamma _L {\hat{k}}_i h_j^N \end{aligned}$$

48 $$\begin{aligned} X^{N \textrm{R}}= &   \lambda _{ij}^N \nabla _i v_j + \gamma _L {\hat{k}}_i h_i^M \end{aligned}$$

with $A_{ij}=\frac{1}{2}\left( \nabla _i v_j+\nabla _j v_i\right)$ and where

49 $$\begin{aligned} \lambda ^N_{ij} = - \frac{1}{2} \lambda ^N ({\hat{l}}_i {\hat{N}}_j + {\hat{l}}_j {\hat{N}}_i) - \frac{1}{2} ({\hat{l}}_i {\hat{N}}_j - {\hat{l}}_j {\hat{N}}_i) \end{aligned}$$

resembles the flow alignment coupling ($\lambda ^N$) and the rotational behavior known from nematic degrees of freedom.

In Eqs. (47) and (48), we have written down explicitly the Larmor-type reversible rotation ($\sim \gamma _L$) of $\delta N$ and ${\hat{k}}_i M_i$. This leads to spin waves, cf. Sect. 3.2, that couple weakly to sound waves (via $\lambda ^N$).

### Reversible currents for $Z_\textrm{A}$

In the $Z_\textrm{A}$ phase, the in-plane preferred direction, ${\hat{Q}}_i$, is even under time reversal. This eliminates the type of reversible currents $\sim \gamma _L$ of the $Z_\textrm{M}$, and only cross-couplings $\sim \lambda _{ij}^N$ are possible

50 $$\begin{aligned} \sigma _{ij}^{\textrm{R}}= &   - \lambda _{ji}^Q \nabla _k h_k^Q \end{aligned}$$

51 $$\begin{aligned} X^{Q \textrm{R}}= &   \lambda _{ij}^Q \nabla _i v_j \end{aligned}$$

with

52 $$\begin{aligned} \lambda ^Q_{ij} = - \frac{1}{2} \lambda ^Q ({\hat{l}}_i ^Q {\hat{Q}}_j + {\hat{l}}_j^Q {\hat{Q}}_i) - \frac{1}{2} ({\hat{l}}_i ^Q {\hat{Q}}_j - {\hat{l}}_j^Q {\hat{Q}}_i) \end{aligned}$$

## Possible experiments

### Static considerations

Clearly, the most interesting static cross-coupling term is the contribution $D_{ijk} (\nabla _i \nabla _j u) (\nabla _k N)$ in Eq. (8), which couples spatial variations of layer compressions to spatial variations of the broken symmetry variable $\delta N$. As an important feature, the contribution $\sim D_{ijk}$ always contains spatial gradients in the three orthogonal directions ${\hat{k}}_i$, ${\hat{N}}_j$ and ${\hat{l}}_k$ of the biaxial system considered here.

To evaluate possible experimental consequences of this contribution, we inspect Eq. (18) for the thermodynamic conjugate associated with spatial variations of $\delta N$, Eq. (3),

53 $$\begin{aligned} h_i^N = L_{ij}^N (\nabla _j N) + D_{jik} (\nabla _k \nabla _j u) \end{aligned}$$

To be specific, we use here (and in the following) a coordinate system for which the layer normal ${\hat{k}}_i$ points into the $z-$ direction and the in-plane preferred direction ${\hat{N}}_i$ is oriented along the $x-$ direction.

Static equilibrium is obtained by $h_i^N = 0$, which requires

54 $$\begin{aligned} \nabla _y N = - \frac{D}{3 L_3^N} \nabla _x \nabla _z u \end{aligned}$$

From Eq. (54), a possible experimental setup to detect effects $\sim D$ arises. Applying a layer compression ($\nabla _z u$) that is tilted in x-direction, a bending of the in-plane direction ($\nabla _y N_y$) within the layers is observed. Since $\nabla _x \nabla _z u$ is externally applied, the conjugate $h_i^u$, Eqs. (14) and (15), does not vanish.

From an experimental point of view, the key challenge appears to be the suppression of the generation of defects in the layering for the spatially varying compression needed. This could be practically achieved by cross-linking and thus generating a gel. Such a system has been investigated previously for usual smectic *A* phases generating a smectic *A* liquid crystalline elastomer [73, 74]. The analysis presented in this Section applies equally well to the antiferroelectric $Z_\textrm{A}$ phase and to the smectic $C_M$ phase.

A possibility to obtain a spatially varying staggered magnetization $N_i$ in $z-$direction would be to apply in-plane gradients of the type $\nabla _x \nabla _y u$. In this case, we have

55 $$\begin{aligned} h_z^N = L_1^N (\nabla _z N) + \frac{D}{3} (\nabla _x \nabla _y u) \end{aligned}$$

which will give rise in static equilibrium to

56 $$\begin{aligned} \nabla _z N = - \frac{D}{3 L_1^N} \nabla _x \nabla _y u \end{aligned}$$

This might be, however, experimentally also quite challenging.

### Sound excitations and spin waves

In solid antiferromagnets, there are spin waves (antiferromagnetic magnons) [50, 51, 75]. In the $Z_\textrm{M}$ phase, similarly antiferromagnetic magnons are found with the dispersion relation $\omega = \omega ({\varvec{q}})$ in harmonic approximation

57 $$\begin{aligned} \omega ^2_m = \gamma _L^2 \chi _{ij}^M {\hat{k}}_i {\hat{k}}_j L_{kl}^N q_k q_l \end{aligned}$$

according to the reversible currents in Eqs. (47) and (48). Note that there is no $\gamma _L$ - type contribution in the $Z_\textrm{A}$ phase (Sect. 2.6), nor in the $C_M$ phase.

Take the layer normal as the $z-$ and the in-plane preferred direction as the $x-$ direction. Equation (57) describes the reversible interplay of $M_z$ with $N_y$ with

58 $$\begin{aligned} L_{kl}^N q_k q_l = L_1^N q_z^2 + L_2^N q_x^2 + L_3^N q_y^2 \equiv L_{q2} \end{aligned}$$

indicating a standard biaxial angular distribution.

In addition, there is, provided by $\lambda _{ij}^N$ in Eq. (45), a reversible coupling to shear and rotational flow (involving $\nabla _x v_y$ and $\nabla _y v_x$) that leads to higher q-contributions

59 $$\begin{aligned} \omega ^2_{m4} = \omega ^2_m \left( 1+ \frac{[\lambda ^N]^2 -1}{4 \rho _0 \gamma _L \chi _{zz}^M} [q_x^2 + q_y^2] \right) \end{aligned}$$

In smectic structures, there is a propagating mode, a layer-compressional wave (often called second sound). To study the influence of static couplings between second sound and spin waves via the contribution $\sim D_{ijk}$ in Eq. (8), we start, first, by assuming incompressibility $(\nabla _i v_i = 0)$ thereby discarding ordinary (first) sound.

Making use of the reversible dynamic equations for the layer displacement *u*, the density of momentum $g_i$, the magnetization $M_i$ and the variable $\delta N$ associated with antiferromagnetism, we arrive at the equations

60 $$\begin{aligned}  &   \dot{N} + \gamma _L \chi _{zz}^M M_z = 0 \end{aligned}$$

61 $$\begin{aligned}  &   \dot{M}_z + \gamma _L \nabla _j ( L_{ij} \nabla _i N + D_{ijk} \nabla _k \nabla _i u) = 0 \end{aligned}$$

62 $$\begin{aligned}  &   \rho _0 \ddot{u} - B \nabla _z^2 u + D_{ijk} \nabla _j \nabla _i \nabla _k N = 0 \end{aligned}$$

Taking a Fourier transform in time and space, we obtain the dispersion relation

63 $$\begin{aligned}  &   \rho _0 \omega ^4 - \omega ^2 (B q_z^2 + \rho _0 \gamma _L^2 \chi _{zz}^M L_{q2} ) + B q_z^2 \gamma _L^2 \chi _{zz}^M L_{q2} \nonumber \\  &   \quad - \gamma _L^2 \chi _{zz} D^2 q_z^2 q_x^2 q_y^2 = 0 \end{aligned}$$

with the abbreviation $L_{q2}$ given in Eq. (58). Neglecting the cross-coupling $\sim D$, we recover immediately the dispersion relations for smectic second sound $\omega _{ss}^2$ and spin waves $\omega _m^2$ in harmonic order

64 $$\begin{aligned} \omega _m^2= &   \gamma _L^2 \chi _{zz}^M L_{q2} \end{aligned}$$

65 $$\begin{aligned} \omega _{ss}^2= &   \frac{B}{\rho _0} q_z^2 \end{aligned}$$

while the coupling term $\sim D$ is of higher order in *q*.

Taking the coupling contributions into account, one gets two coupled modes

66 $$\begin{aligned} 2 \omega _{1,2}^2= &   \omega _m^2 + \omega _{ss}^2 \pm \sqrt{(\omega _m^2 - \omega _{ss}^2)^2 + 4 R} \end{aligned}$$

with $R= (1/\rho _0) \chi _{zz} \gamma _L^2 D^2 q_z^2 q_x^2 q_y^2$. From the inspection of the expression for *R*, it is clear that this coupling only occurs, when the wave vector ${\varvec{q}}$ is oblique to all three preferred directions.

The dissipation of $\dot{N}$ ($\sim 1/\gamma ^N$) and that of $\dot{M}_z$ ($\sim b_{ijkl}$) in Eq. (34) enter the dispersion relation, Eq. (66), in order $\omega ^2 \sim q^3$, which is in the same order as the coupling $\sqrt{R}$.

### Selected dissipative dynamic aspects

There is a dissipative cross-coupling between gradients of an electric field, $\nabla _i E_j$ and the in-plane degree of freedom, $\delta N$. In the dissipation function, Eq. (34), this can be described by the additional contribution

67 $$\begin{aligned} R^E= &   \psi _{ij}^N (\nabla _i E_j) (\nabla _k h^N_k) \end{aligned}$$

where $\psi _{ij}^N$ takes under the assumption $\mathbf{\nabla \times E \equiv 0}$ the form

68 $$\begin{aligned} \psi _{ij}^N= &   \psi ^N ({\hat{N}}_i {\hat{l}}_j + {\hat{N}}_j {\hat{l}}_i) \end{aligned}$$

As a result, the dissipative quasi-current $X^{N \textrm{D}}$, Eq. (39), acquires an additional contribution and reads

69 $$\begin{aligned} X^{N \textrm{D}} = - \frac{1}{\gamma ^N} \nabla _i h^N_i - \psi _{ij}^N \nabla _i E_j \end{aligned}$$

Generally, an external inhomogeneous electric field will take the (highly coupled) system of macroscopic degrees of freedom into a dynamic state. However, after some transition period, dissipation will drive the system toward a (final) stationary state with $X^{N \textrm{D}} = 0$.

Taking again the layer normal as the $z-$ and ${\hat{N}}_i$ as the $x-$ direction, we obtain a vanishing quasi-current for

70 $$\begin{aligned} (L_1^N \nabla _z^2 + L_2^N \nabla _x^2 + L_3^N \nabla _y^2) N = - \gamma ^N \psi ^N \nabla _x E_y \end{aligned}$$

From Eq. (70), we read off immediately that gradients of an electric field applied in the layer planes of the $Z_\textrm{M}$ phase give rise to spatial variations of the in-plane direction. In general, these spatial variations will involve all three orthogonal directions of this biaxial phase. We also note that in the $Z_\textrm{A}$ (and the $C_M$) phase the same type of effects is possible.

There is no dissipative cross-coupling between gradients of a magnetic field, $\nabla _i H_j$ and the in-plane degree of freedom in the $Z_\textrm{M}$, $Z_\textrm{A}$ and $C_M$ phase.

## On the question of antiferroelectricity and antiferromagnetism in nematic liquid crystals

Antiferromagnetism and antiferroelectricity are well-established subfields of solid-state physics. In antiferromagnets, spins of opposite sign are occupying different sublattices in three dimensions (3D) in a way such that there is no net magnetization left over in the bulk. This type of order is frequently described by the introduction of the concept of a staggered magnetization for which one turns around the spin projection of on one of the sublattices. Antiferroelectrics are the electric analog of antiferromagnets in the sense that charges of opposite sign are occupying different sublattices in 3D in such a way that there is no spontaneous electric polarization. In both types of systems, the building blocks (atoms, ions, molecules) are positionally ordered in 3D.

Here, we contrast this situation with the case of nematic liquid crystals, which do not show any long-range positional order in 3D. Instead, they are characterized by one or two preferred directions (in the uniaxial and biaxial case, respectively) that show long-range orientational order.

Over the last decade or so the question of the simultaneous presence of orientational long-range order in the electric and/or magnetic domain has attracted increasing attention. By now ferromagnetic, as well as ferroelectric, nematic liquid crystals are well established experimentally and have also been described macroscopically theoretically. In both cases, the spontaneous magnetization or the spontaneous electric polarization is reported to be parallel to the uniaxial nematic director field. Simultaneously, the positional order in ferromagnetic nematics as well as in ferroelectric nematics is still short range in 3D.

Rather recently the question of long-range order of the macroscopic polarization in smectic (layered) liquid crystals has come into the focus of experimental investigations. As of today both, ferroelectric as well as antiferroelectric liquid crystalline phases have been obtained and described experimentally for the ordinary type of smectic *A* liquid crystalline phases. Here, layers are positionally ordered along a preferred direction, the layer normal. Within the layers no preferred directions are present. In ferroelectric smectic *A* phases, denoted as $A_F$ the macroscopic polarization is oriented parallel to the layer normal. In contrast, for antiferroelectric smectic liquid crystalline phases, called $Z_\textrm{A}$ in the literature, the electric polarization is found to be in a certain direction in the layer planes alternating from one layer to the next: In one layer, it can be denoted as $+$ and in the next layer with −, thus giving rise to vanishing overall electric polarization. Then, one can define a staggered polarization as is formulated (for the magnetic case) in Eqs. (1) - (3).

Apparently, one needs a macroscale structure (interface) that separates the two different polarities, in order to allow for antiferro behavior. In solid antiferroelectrics, this is provided by regular arrangements of spatial building blocks, in smectics by the layer structure. This immediately answers the question, why are there no antiferroelectric nematic liquid crystals? Because, by definition, there are no regular spatially ordered structures in nematics (with 3D positional disorder). And without spatially ordered structures, the polar building blocks (typically organic molecules) are mixed and disordered in all three spatial dimensions on molecular length scales. We therefore conclude that the $N_x$ phase reported on Refs. [9] and [76] cannot be an antiferroelectric nematic phase, but is most likely an antiferroelectric smectic phase called $Z_\textrm{A}$ in the literature.

A few remarks are in order.

First, there is no problem with antiferroelectricity in columnar liquid crystalline phases, since these show 2D positional order. But no such phases have been reported until now.

Second, the analysis given above for antiferroelectricity applies equally well to *ferri*electricity with only partly compensated electric order. In particular, it cannot occur in nematic liquid crystals due to the lack of any positional long-range order.

Third, symmetry would allow in smectics an additional ferroelectric phase, where the (homogeneous) polarization lies in the layers and an additional antiferroelectric phase with the staggered magnetization along the layer normal.

Forth, what has been discussed above for the electric case applies also to the magnetic case, although ferromagnetic and antiferromagnetic phases have not yet been found for smectic *A* liquid crystals.

## Defects of the in-plane preferred direction in smectic $Z_\textrm{A}$

It is well known that defects in ordinary uniaxial nematics have half-integer strength. This is due to the invariance of ${\hat{n}}_i \rightarrow - {\hat{n}}_i$ ($I_n$): After a $180^o$ degree rotation, the original state is regained, thus the half-integer defect strength.

For the smectic $C_M$ phase, with $D_{2h}$ symmetry (orthorhombic and non-polar), it has been predicted [45] and found later experimentally [77] that defects in the in-plane rotation angle, $\delta m$, can be of half-integer strength 1/2, due to the $I_m$ invariance. The same arguments apply for the $Z_\textrm{A}$ and the $Z_\textrm{M}$ phases, since the in-plane preferred directions, ${\hat{Q}}_i$ and ${\hat{N}}_i$, are subject to the $I_Q$ and $I_N$ invariance.

This is in contrast to the usual tilted smectic *C* phase, which has a lower symmetry ($C_{2h}$, monoclinic). It allows only for defects of integer strength in the in-plane director, ${\hat{c}}_i$, since it does not have a ${\hat{c}}_i \rightarrow - {\hat{c}}_i$ symmetry. (There is only a combined symmetry ${\hat{c}}_i \rightarrow - {\hat{c}}_i$ together with ${\hat{k}}_i \rightarrow - {\hat{k}}_i$.) Similarly, polar nematics, $N_F$, ferromagnetic nematics, $N_A$, as well as ferroelectric and ferromagnetic smectic phases, $A_F$ or $A_M$, have integer defects, because there is no $P_i$ to $- P_i$ nor a $M_i$ to $- M_i$ symmetry.

In Ref. [9], defects of half-integer strength have been observed for the usual nematic phase and, remarkably, for the phase called $N_x$ (referring to Figure S2 of the Supporting Information of Ref. [9]), while only defects of integer strength have been observed for the ferroelectric phase $N_F$. Our symmetry analysis rules out that the $N_x$ phase is a nematic phase. We would like to emphasize that the precise nature of the $N_x$ phase was unknown, when Ref. [9] was written and this phase is called smectic $Z_\textrm{A}$ today. We note that the observation of half-integer defects has also been confirmed experimentally very recently for the $Z_\textrm{A}$ phases analyzed by Nacke et al. [5].

## Summary and perspective

In this work, we have presented the macroscopic dynamics of antiferroelectric smectic $Z_\textrm{A}$ and antiferromagnetic smectic $Z_\textrm{M}$ liquid crystals. The experimentally observed smectic $Z_\textrm{A}$ phase is characterized by antiferroelectric order in one direction in the planes of the smectic layers giving rise to an orthogonal biaxial overall symmetry without polar direction in sufficiently thick samples.

In addition, we have investigated the antiferromagnetic analog of the smectic $Z_\textrm{A}$ phase, which we denoted as $Z_\textrm{M}$ and which has as yet to be found experimentally. In $Z_\textrm{M}$, one also has an in-plane preferred direction, which is, however, not like a director in an ordinary nematic, but odd under time reversal. When compared to usual non-polar smectic *A* phases, the additional macroscopic variables are an in-plane staggered magnetization ${\varvec{N}}$ and the magnetization ${\varvec{M}}$. Thus, spin waves become possible and there are three pairs of propagating modes: first and ‘second’ sound as in usual smectic *A* phases and a pair of spin waves. We find that the coupling between ‘second’ sound and spin waves is rather weak, since it is a higher order effect in the wave vector *q*.

It will be quite interesting to study how the antiferroelectric nature of the smectic $Z_\textrm{A}$ is modified in a freely suspended film when the number of layers is reduced say below 10 layers. So far freely suspended films of $Z_\textrm{A}$ have apparently not been investigated for the $Z_\textrm{A}$ phase.

We have also analyzed the question of antiferroelectricity and antiferromagnetism for nematic liquid crystals. As a result, we find that both, antiferroelectricity as well as antiferromagnetism cannot exist in nematic liquid crystals, since they are positionally disordered in all three spatial dimensions. The same applies to ferrimagnetism and ferrielectricity for which one has partially compensated sublattices in a solid.

There are several directions into which one can generalize the analysis presented in this paper. First of all one could produce a material composed of chiral molecules, which will most likely lead to antiferroelectric or antiferromagnetic smectic phases, which will also break mirror symmetry in all three spatial dimensions, in contrast to the smectic $$Z_A $$ and $$Z_\textrm{M}$$ phases analyzed here.

Since both antiferroelectricity and antiferromagnetism in smectics require an exact compensation of the electric polarization or the magnetization on alternating layers, it will be rather interesting to study the influence of a network leading to antiferroelectric or antiferromagnetic smectic elastomers and gels. So far, such a combination has apparently not yet been attempted experimentally. In particular, it would be important to check whether an anisotropic network could lead to the destruction of antiferroelectricity and/or antiferromagnetism,

Another direction to go into would be the investigation of the question of antiferroelectricity and antiferromagnetism for columnar liquid crystals. These show typically positional order in two dimensions and frequently one-dimensional fluid order in the third dimension. Thus, one can easily envisage several two-dimensional lattices for which one has an exact compensation of the electric polarization or magnetization in adjacent columns. We just mention a square lattice as one possibility.

## Data Availability

Data sharing is not applicable to this article.
